# Feeding preferences of the Asian elephant (*Elephas maximus*) in Nepal

**DOI:** 10.1186/s12898-016-0105-9

**Published:** 2016-11-17

**Authors:** Raj Kumar Koirala, David Raubenheimer, Achyut Aryal, Mitra Lal Pathak, Weihong Ji

**Affiliations:** 1 Human Wildlife Interaction Research Group, Institute of Natural and Mathematical Sciences, Massey University, Private Bag 102 904, Albany, Auckland, 0745 New Zealand; 2 Institute of Forestry, Tribhuvan University, Pokhara, Nepal; 3 The Charles Perkins Centre and School of Life and Environmental Sciences, University of Sydney, Sydney, NSW Australia; 4 Department of Forestry & Resource Management, Toi Ohomai Institute of Technology , Rotorua, 3046 New Zealand; 5 Waste Management NZ Ltd, Auckland, New Zealand; 6 Department of Plant Resources, National Herbarium and Plant Laboratories, Godawari, Nepal

**Keywords:** Browse. elephant habitat, Feeding sign, Food preferences, Micro-histological analysis

## Abstract

**Background:**

Nepal provides habitat for approximately 100–125 wild Asian elephants (*Elephas maximus*). Although a small proportion of the world population of this species, this group is important for maintaining the genetic diversity of elephants and conservation of biodiversity in this region. Knowledge of foraging patterns of these animals, which is important for understanding their habitat requirements and for assessing their habitat condition, is lacking for the main areas populated by elephants in Nepal. This study investigates the feeding preferences of the Asian elephant in Parsa Wildlife Reserve (PWR) and Chitwan National Park (CNP), Nepal.

**Result:**

Fifty-seven species of plants in 25 families were found to be eaten by Asian elephants, including 12 species of grasses, five shrubs, two climbers, one herb and 37 species of trees. The species that contributed the greatest proportion of the elephant’s diet were *Spatholobus parviflorus* (20.2%), *Saccharum spontaneum* (7.1%), *Shorea robusta* (6.3), *Mallotus philippensis* (5.7%), *Garuga pinnata* (4.3%). *Saccharum bengalensis* (4.2%)*, Cymbopogan* spp (3.7%), *Litsea monopetala* (3.6) and *Phoenix humilis* (2.9%). The preference index (PI) showed that browsed species were preferred during the dry season, while browsed species and grasses were both important food sources during the rainy season. Elephants targeted leaves and twigs more than other parts of plants (*P* < 0.05).

**Conclusion:**

This study presents useful information on foraging patterns and baseline data for elephant habitat management in the PWR and CNP in the south central region of Nepal.

**Electronic supplementary material:**

The online version of this article (doi:10.1186/s12898-016-0105-9) contains supplementary material, which is available to authorized users.

## Background

Elephants are among the internationally endangered large mammals [1]. The habitat of Asian elephants (*Elephas maximus)* has been decreasing throughout their range, due primarily to habitat destruction and fragmentation resulting from human land use practices [2, 3]. Even though elephant populations have decreased, in general, the local density of elephants has increased due to habitat loss [4]. This has caused resource competition among elephants [5], and increased human–elephant conflict [6]. Asian elephants are generalised herbivores utilising a variety of plant species [2, 7]. Large herbivores such as elephants require extensive home ranges to satisfy their high food demand [8]. Reduction in food availability due to loss of habitat has created challenges for elephant conservation in the many regions in Asia.

Although the dietary requirements of Asian elephants have been studied, the majority of these studies [2, 5, 9, 10] have dealt with the documentation of food plant species, the rate of consumption and seasonal comparative diet overlap between sympatric elephants and rhinos [11, 12]. However, details regarding food choice and seasonal diet composition remain unknown. Such information is important for Asian elephant conservation in terms of habitat management and human–elephant conflict mitigation.

Nepal provides important habitat for Asian elephants. Historically habitat in the Terai range was continuous. Currently, elephants are found only in four regions of the country, eastern, central, western and far-western. In central Nepal, Parsa Wildlife Reserve (PWR) is the main elephant habitat. However, elephants were found to migrate between PWR and Chitwan National Park (CNP) since the middle of the 1990s [13]. The migration of elephants between these sites was thought to be primarily due to the reduction of water availability in the Bara Forest near PWR resulting in reduced food availability and aggravated competition with livestock [13]. Currently, all the four isolated population of elephants in Nepal are in the lowland Terai region. These widespread and fragmentary distributed elephants strongly prefer floodplain communities, and there is a significant shift from browse to grass-dominated vegetation between seasons in Bardia National Park [12, 14]. However, the diet has not been studied for other elephant populations of the country.

This study aims to investigate the food preferences and seasonal changes in foraging patterns of the Asian elephant in the PWR, CNP and adjoining forests. We predict a climate-related reduction in grass productivity in the Parsa area will correspond with a reduction of grass in the elephant diet during the dry season. Information obtained from this study will aid elephant conservation in respect to the restoration of their habitats, and will thereby contribute towards minimising human–elephant conflict.

## Methods

**Fig. 1:**
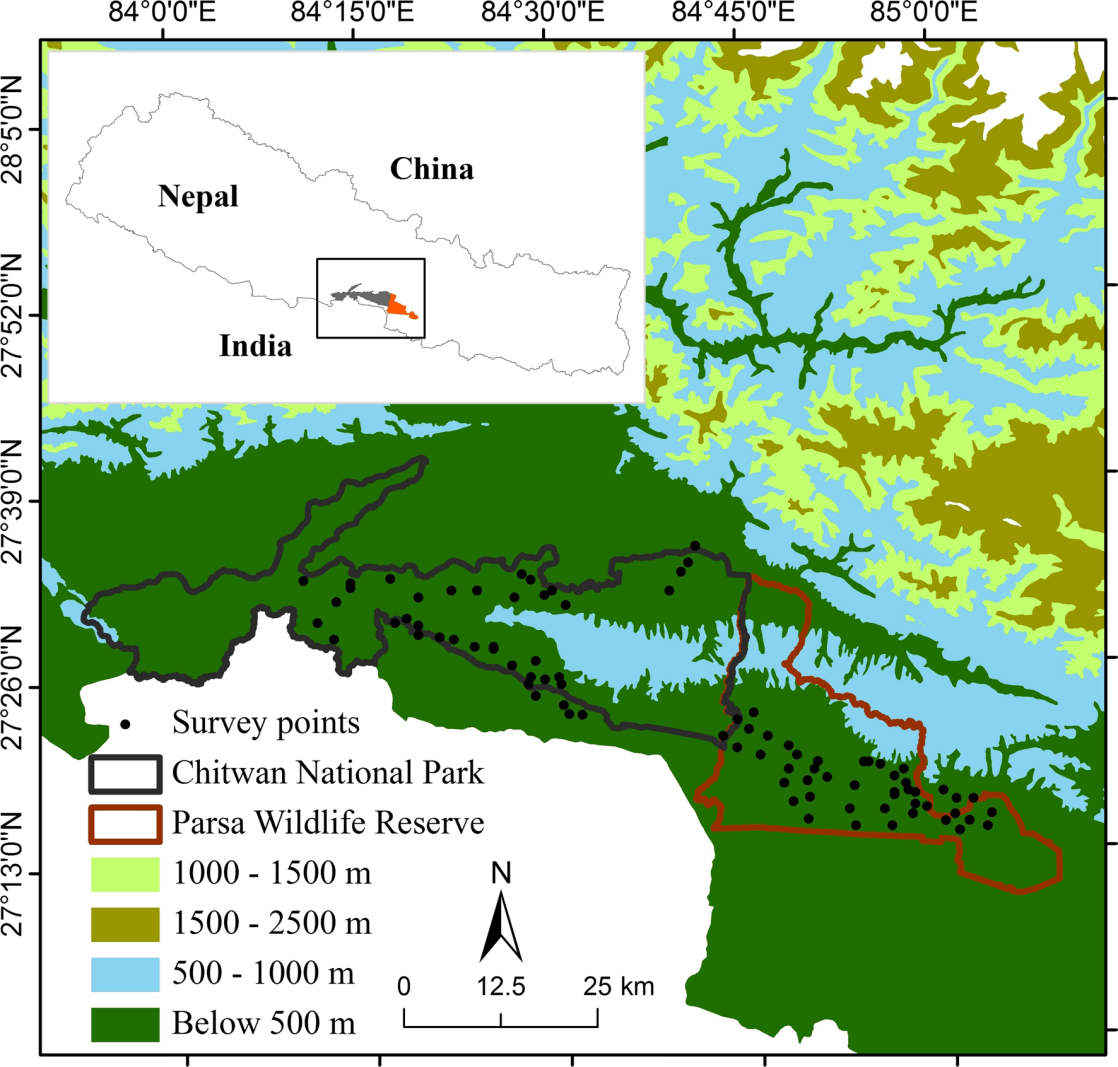
Map of the Parsa Wildlife Reserve (PWR) and Chitwan National Park (CNP) showing locations of plots used for vegetation and feeding sign surveys

The study was carried out at the Parsa Wildlife Reserve and part of adjoining reserve forest (Bara forest) in the north and Chitwan National Park and part of its buffer Zone forests. Permission for the study was acquired from the Department of National Park and Wildlife Conservation, the government of Nepal. Parsa Wildlife Reserve is the largest wildlife reserve in Nepal (Fig. 1), consisting of 499 km^2^ sub-tropical forests in the south-central lowland Terai ecoregion of Nepal. The PWR is located in the Churia hills, the outermost foothills of the Himalayas [15], which are a part of the Bhabar District. The PWR is typically dry with average rainfall between 300 and 450 mm during the summer months [13, 16]. The typical vegetation of this reserve and the adjoining Bara forest is tropical and subtropical forest types with Sal (*Shorea robusta*) forest about 90% of the vegetation. Chirpine (*Pinus roxburghii*) grows in the Churia hills. Khair (*Acacia catechu*), Sisso (*Dalbergia sisso*) and Silk cotton (*Bombax ceiba*) trees occur along water channel. Sabai grass (*Eulaliopsis binata*) grows well on the southern face of the Churia Hills [17, 18]. Chitwan National Park was established in 1973 as the first national park in Nepal and was listed as a World Heritage Site in 1984. The CNP spans 932 km^2^ and is situated in the sub-tropical lowlands of the Inner Terai, in the Chitwan district of south-central Nepal (Fig. 1). Elevation ranges from approximately 100 m in lowland river valleys to 815 m on Churia Hill ridgetops. In the north-west of this protected area, the Narayani and Rapti rivers separate the park from human settlements [19]. The buffer zone has mostly agriculture fields and human settlements along with community forests. The typical vegetation of CNP and its buffer zone forests is Himalayan subtropical broadleaf forests with primarily Sal (*Shorea robusta*) trees covering about 70% of the national park area. On northern slopes, Sal associated with smaller flowering tree and shrub species such as *Terminalia bellirica, Dalbergia sissoo, Dillenia indica, Garuga pinnata* and climbers such as *Bauhinia vahlii* and *Spatholobus parviflorus* [17, 18, 20].

Both the PWR and CNP are prime habitats for wild Asian elephants and both parks are adjacent to Valmiki tiger reserve in India (Fig. 1). These three trans-boundary, contiguous protected areas cover a 3549 km^2^ mixed-habitat zone containing large tracts of grasslands and humid deciduous forests, which provide suitable habitat for a large number of megaherbivores and big cats such as Asian elephants, endangered tigers (*Panthera tigris*) and greater one-horned rhinos (*Rhinoceros unicornis*).

### Elephant feeding data collection

Opportunistic direct feeding observations and the observation of elephant feeding sign on food trails (elephant feeding routes) were the methods used in the present study to determine diet selection of elephants residing in different areas and travelling on different migration routes [9, 21]. The feeding routes observed to be taken by elephants were followed by field researchers, and all plant species showing signs of being eaten by elephants were recorded. Evidence of feeding sign included elephant footprints, fresh dung piles nearby to browsed foliage, and the identifying characteristics of plant damage caused by elephant browse, such as debarkation, branch breaking and uprooting. The following data were recorded to determine the feeding preferences of Asian elephants: (1) plant species browsed, (2) parts of the plant eaten (leaves, branches and/or bark), (3) habitat type and (4) global positioning system (GPS) coordinates of sample sites (Fig. 1). The relative frequency (percentage) of feeding sign was calculated to yield a feeding sign score. Feeding sign was ranked according to the intensity of browsing, the proportion of bark, stem and foliage removed and/or the area of grass eaten.

### Elephant dietary analysis from dung samples

Samples of elephant dung encountered during a total 24 days of field survey in the wet season (June–September 2012) and the dry season (February–April 2013) were collected. Visual examination of deposited elephant dung piles was performed to identify the presence of macro plant fragments. Micro-plant fragments were identified through micro-histological analysis [22–24]. This dual methodology is widely used for estimating the diet composition of herbivores [25]. Fragments of probable food species were collected for the preparation of reference slides. The collection was made as per methods used in the previously published literature describing elephant food plants [11, 12]. A total of 20, non-overlapping random fragments were isolated on each dung slide and were compared with a reference slide for epidermal derivatives. Microphotographs were taken using a 100 × 4× lens and an Am Scope MT130 1.3 megapixel USB2.0 microscope eyepiece digital camera.

### Food availability survey

**Fig. 2:**
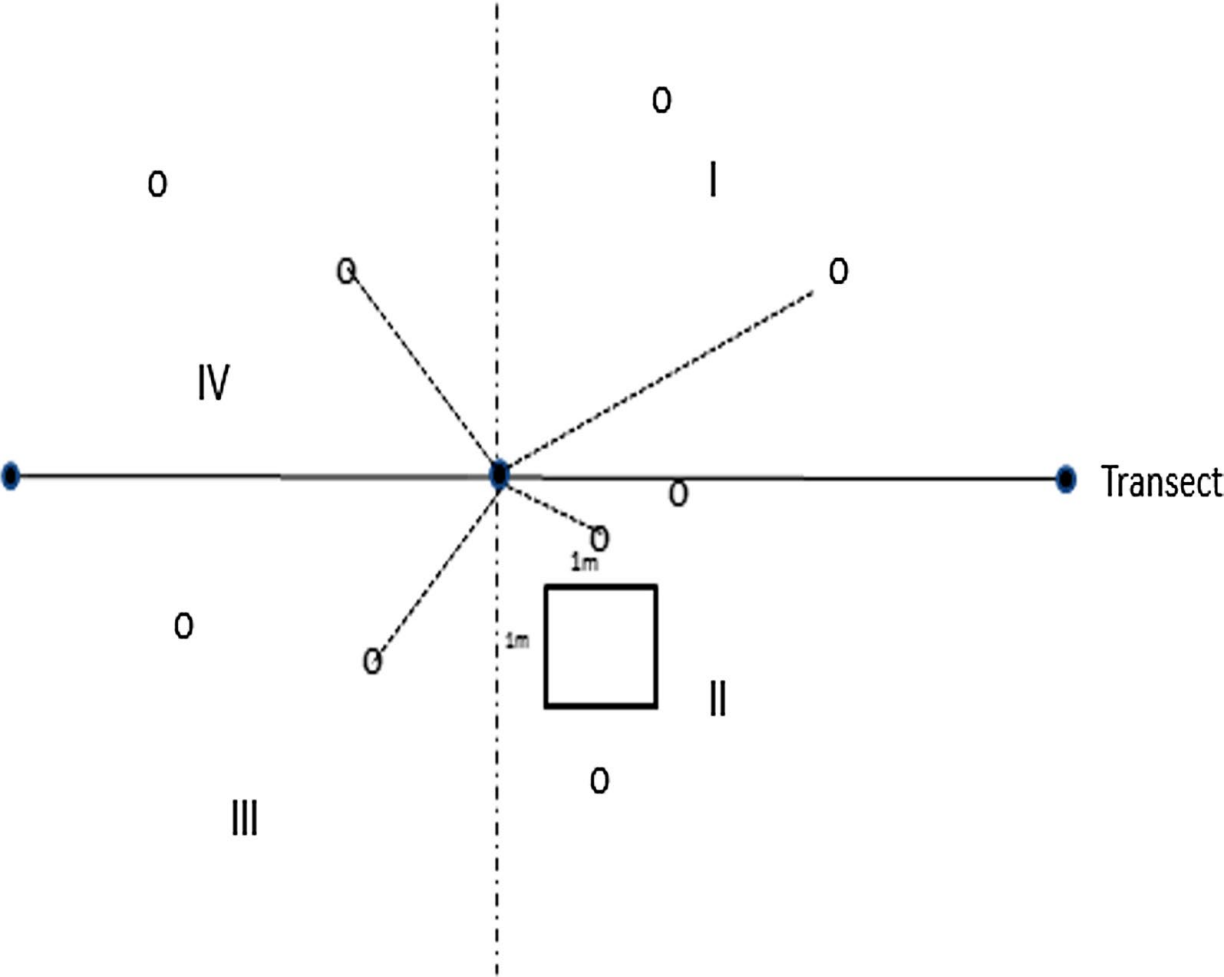
Sample points along a transect with the nearest trees in each quarter indicated by *dash lines* and a grass of 1 m × 1 m near each sample points

To assess the food preferences of elephants, we carried out vegetation surveys using the point-centred quarter technique [26] to obtain data on the relative abundance of different plant species. A total of 30 transects of 2 km length each, one each per habitat type, were created for this survey. To compare the availability of food plants within and outside protected areas, 20 of these transects were placed in the protected areas, while the remaining ten transects were located in habitats outside national parks. Each transect was surveyed twice, once in the wet season (August/September) and once in the dry season (March/April). A total of 10 sample points were assigned to each transect at 200 m interval for the purpose of gathering data on potential forage trees. Also, 10 quadrats measuring 1 m × 1 m each were created near each sample points to collect data on density, frequency and visual estimation of cover % of dietary grass species. At each sample point, a cross was laid on the ground to divide the area into four quarters (Fig. 2). From each quarter, the closest tree from the centre was identified and the following data collected: (1) the species of the tree, (2) the distance from the tree to the centre of the quarter, (3) diameter at breast height (DBH) of the tree.

### Data analysis

Elephant feeding sign survey was conducted by scoring the different signs according to the intensity of damage. The definition of scores for bark was: 1 ≤ 0.5 m^2^; 2 = 0.5–1 m^2^; 3 ≥ 1 m^2^; for branch score: 1 = up to 5 cm diameter; 2 = 5–20; 3 > 20; while foliage score: 1 ≤ 10% of foliage eaten; 2 = 10–40%, 3 ≥ 40% and grass score of 1 = up to 1 m diameter; 2 = more than 2 m; 3 = more than 5 m. Total feeding score was calculated by multiplying the frequency of each plant species showing feeding signs with total feeding sign score of that species. Total feeding score of each species was multiplied by 100 and divided by the total feeding score of all species to calculate an index equivalent to utilisation percent. The importance value index (IVI) of a plant species in each habitat was calculated by adding the relative frequency, density and dominance (basal area) for trees. The relative frequency, density and cover for grass and herbaceous species was used as an index of availability of a species in the study area [21]. The density of browse was calculated using the distance from the tree to the centre of the quarter following Mitchell [27].

The preference index (PI) was calculated using the following equation [21, 28]:

1 $${\text{Preference}}\;{\text{index (PI) = }}\frac{\text{Utilization percentage }}{\text{Percentage availability in the environment}}$$

A PI score >1 indicates food that was utilised proportionately more than its occurrence in the environment, and a PI score <1 indicates a plant food that was used proportionately less than its presence in the environment.

The Chi-square test was used to test for differences in feeding preferences between plant parts, seasons and sites differences in vegetation density; Pearson correlation was used to determine the correlation between forage availability and preference. Simpson’s diversity index was used to estimate the vegetation diversity, and the independent sample *t* test was applied to test for seasonal dietary intake differences in monocot and dicot plants. All tests were performed using Excel and IBM SPSS statistical version 22.

## Results

### Elephant foraging patterns

In total, 57 species of plants (13 grass, five shrubs, two climber, one herb and 36 tree species) belonging to 27 taxonomic families were eaten by Asian elephants. In the Parsa area, 40 species (10 grass, four shrub, two climber, one herb and 23 tree species) were consumed, and in the Chitwan area 37 species (nine grass, three shrub, one climber and 24 tree species) were utilised; the utilisation pattern suggests that 76% of all identified food species were consumed during the wet season, with only 24% consumed during the dry season (Additional file 1: Appendix). The foliage (leaves and twigs) of both grasses and browsed trees were selected more than the stems, bark, roots and fruits during the wet season in both Parsa and Chitwan (χ^2^ = 10.72, df = 6, P < 0.05), whereas debarkation and uprooting were more common in the dry season (χ^2^ = 5.24, df = 4, P < 0.05).

### Dietary analysis from dung samples

Microscopic analysis of 36 dung samples collected during two seasons showed a higher dicot-to-monocot ratio in the dry season compared to the wet season. The average dicot-to-monocot ratio was 1:0.57 in the dry season, whereas the ratio was 1:1.11 in the rainy season. The observations from the feeding sign survey and the micro-histological analysis revealed that dicots were consumed more during the dry season (t = −4.27, df = 10, P = 0.002). There was no significant difference in the presence of dicot and monocot plants in elephant diet during the rainy season (t = 1.59, df = 58, P = 0.117).

### Regional food availability

There was no difference in the types of plants availability in and outside the two sites (P ≥ 0.05). However, species diversity was slightly lower in CNP (Simpson’s diversity index, D = 0.097) than in the PWR (D = 0.091). Similarly, in both study sites and seasons, food species densities and frequencies recorded were significantly different.

There was a significant relationship of grass and browse abundance in dry and wet season in Parsa and Chitwan, indicating an association between these factors (χ^2^ = 8.92, df = 1, P = 0.002). Higher densities of each browse species were recorded in the PWR (mean density, 25.00/ha) than in the CNP (mean density, 20.4/ha). Seasonally the wet season mean density of each browse species in Chitwan and Parsa were 23.2 and 15.4/ha respectively. In the dry season, the mean density of browse in Chitwan was 16.3 and in Parsa 20.0/ha. There was significant difference in the frequency of grasses (χ2 = 20, df = 1, P < 0.001) in the dry season in both parks with higher frequencies of grass species recorded in the dry season in CNP (mean frequency 3.45/q; mean density, 115.7/m^2^) than in PWR (mean frequency, 1.57/q; mean density, 22.85/m^2^). The mean grass frequency and density in the wet season in Chitwan was 4.5/q and 160 individuals/m^2^, respectively. In Parsa, the mean grass frequency and density was 2.0/q and 131 individuals/m^2^. There was a negative correlation between the availability of individual plant food species in the habitat and their utilisation by elephants (r = −0.244, P = 0.02).

### Plant species preferences

**Fig. 3:**
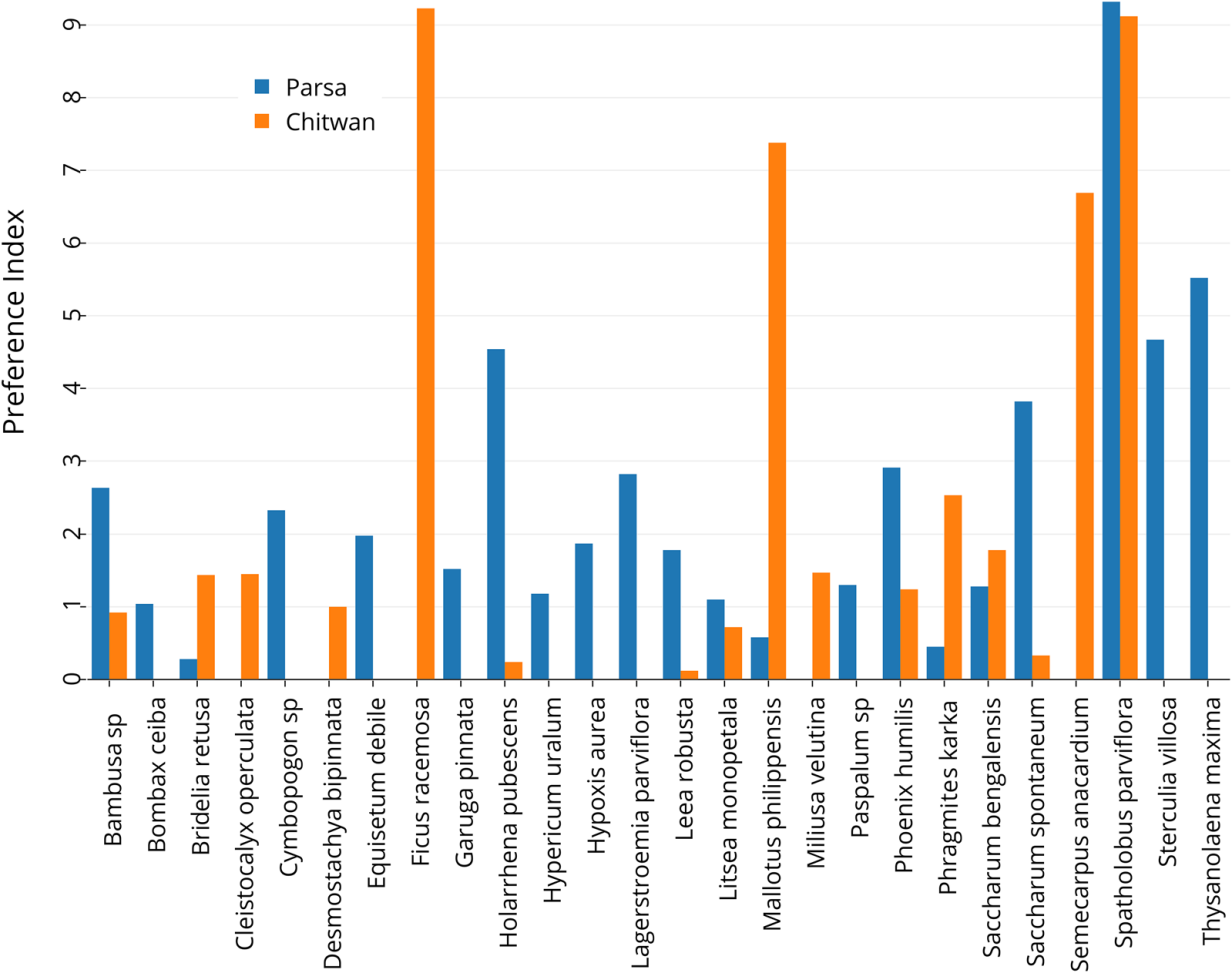
Preference indices (PI) for the most prevalent plant species found in the diet of wild Asian elephants in the Chitwan National Park (CNP) and the Parsa Wildlife Reserve (PWR) in both the rainy and dry seasons

Elephants showed a positive PI score for 26 out of the 57 utilised plant species (Fig. 3). Elephant browse that had relatively high PI scores ranged from 1.04 (*Bombax ceiba*) to 9.2 (*Ficus racemosa*). Similarly, vine PI scores ranged from 0.02 (*Bauhinia vahlii*) to 9.32 (*Spatholobus parviflorus*). Shrubs that had relatively high PI scores were *Hypericum uralum* (1.18) and the palm *Phoenix humilis* (2.91). Grass PI scores ranged from 1.28 (*Saccharum bengalensis)* to 5.51 (*Thysanolaena maxima*). Species that were highly abundant, which may have led to lower PI scores, included *Shorea robusta, Dillenia pentagyna, Hemarthria compressa, Imperata cylindrica* and *Cymbopogon* spp.

Overall, in both sites, elephants showed the strongest preferences for common species such as *Spatholobus parviflorus, Saccharum spontaneum, Phoenix humilis, Saccharum bengalensis, Mallotus philippensis,* and *Phragmites karka*. In addition to these species, elephants in the Chitwan area showed a strong preference for *Cleistocalyx operculata* and *Bridelia retusa*, while Parsa area elephants showed a strong preference for *Litsea monopetala*, *Thysanolaena maxima*, *Sterculia villosa, Equisetum debile, Bambusa* spp. and *Hypericum uralum*. The availability of these species in the two parks varies. Amongst these 26 most preferred species, 17 species were preferred more by elephants in Parsa, while the remainder (nine species) were preferred relatively more by Chitwan elephants (Fig. 3).

## Discussion

Nepal has lost over 80% of its elephant habitat to human settlement [5]. As a result, the resident elephant population, estimated to number between 109 and 142 individuals, is presently restricted to four isolated areas [29]. Available diet and nutritional preference are the two most important factors that drive elephant movements, and that generate conflict with humans, especially when available elephant habitat is shrinking [30]. Reduction in grass, especially in the dry seasons may result in elephant migration. Human–elephant conflicts may arise mainly due to elephant migration [31]. Thus, knowledge of elephant foraging patterns and seasonal food availability is important for mitigation of human–elephant conflict. The management of grass species in the dry season is crucial. In areas like Parsa where there is an environmental constraint in retaining surface water, some potential habitats could be irrigated during the dry season to maintain grass productivity.

The present study recorded 57 plant species within 25 families that were foraged by Asian elephants in the PWR and CNP. In a similar study, Sukumar [2] reported 112 species of plants in the elephant's diet in southern India, and Chen et al. [9] reported 106 plant species in the diets of elephants in Shangyong National Natural Reserve in Xishuangbanna, the People’s Republic of China were catalogued. The wide range of results between studies may be due to differences in the number and diversity of plant species available. Divergent results may also be partly due to differences in sampling methods; variances in forest condition (disturbed versus undisturbed), composition, and sampling area could also have contributed to divergent results.

Elephants are mixed feeders, and there is seasonal variation in their food selection [8]. In the present study, we found that browse flora and grasses were both eaten by elephants during the wet season, while browse vegetation dominated the dry season diet. Indeed, it seems that the proportion of dicot and monocot species in the diet of elephants varies across different home ranges. In southern India, elephants are known to rely heavily on graminoids (grasses, sedges and rushes) in the wet season and almost exclusively on woody plants during the dry season [32]. Similar patterns of seasonal variation in feeding have been reported by Pradhan et al. [12] in Bardia National Park in Nepal, and also for African elephant in Uganda [33]. In Nilgiri Biosphere Reserve, southern India, grasses dominate the elephant diet in all seasons, while browse flora forms an important portion of their diet only during the dry season [10]. Likewise, in the foothills of the Himalayas, browse forms the majority of the diet in dry seasons [34]. In similar studies, browse dominated the diet of elephants all year in the rainforests of Malaysia [35], north-eastern India [36] and in the state of Bihar, Central India [37].

Results of the present study are comparable to the data obtained in the above-mentioned studies in terms of dry-season diet. This browse-dominated dry season diet could be due to the lower average grass biomass available when the dry season causes a reduction in grass cover. It could also be due to the need to meet specific nutrient requirements, for example, the high levels of essential minerals in the hard wood of browse plants [12]. However, our study revealed a slightly different trend in the wet season, when a similar proportion of grass and browse were found in the elephant diets. This could be due to the migration patterns of elephants in Nepal: at the onset of the rainy monsoon season, elephants move from Chitwan to Parsa and towards upper slopes [13]. As the monsoon develops, elephants migrate from grass-rich lower elevations south to the foothills of the Churia range for occasional resting. In the Churia foothills, elephants have more opportunities to eat foods other than grasses, as these foothills are rich in preferred woody species.

In the present study, we noted a difference in feeding preference for stems, leaves and twigs, bark and other parts of woody plant species. Foliage (leaves and twigs) of both grass and browse flora were eaten more than other parts of plants in the wet season, while bark dominated the dry season diet. The use of bark from various tree species by elephants might relate to macronutrient balancing [38], and for gaining moisture and mineral supplements [39] that would otherwise have been unavailable during the dry season. The current study aligns with the findings of Pradhan et al. [12] in Bardia National Park, Nepal, where bark consumption dominated the diet of elephants in the dry season. Differences in forest structure, methodologies used and spatial and temporal availability of different groups of plants could explain the variance in PI between the two studies, which are both based on elephant populations in Nepal.

Spatially and temporally, PI can vary between species. In the present study, widely abundant foods such as *Shorea robusta, Mallotus philippensis, Imperata cylindrica* and *Saccharum bengalensis* were avoided by elephants in some seasons and locations, despite their high availability (Additional file 1: Appendix). Therefore, it is important to examine independently the PI scores of species that are of high availability (or rare) to determine whether the score could be due to the methodological limitations of this index alone [40], or could involve other factors. The PI scores derived from Parsa and Chitwan could be obtained from multiple rather than single factors [41]. Factors such as seasonal availability [42], palatability [43], nutritive value and plant tissue toxicity are all important influences on the selection of food plants by elephants [35].

Although in both the PWR and the CNP, elephants prefer common plants such as *Spatholobus parviflorus,* in fact* Saccarum spontaneum, Phoenix humilis, Saccharum bengalensis*,* Mallotus philippensis* and *Phragmites karka*, there are some less common species such as *Acacia catechu, Bombax ceiba, Bamboosa* spp and *Ficus* spp that are important food for elephants. In the present study, feeding patterns observed in both areas revealed that Parsa elephants ate a more diverse, species-rich diet than did Chitwan’s Asian elephant population. The Parsa area has a higher number of elephants, possibly suggesting that nutrition is superior in PWR due to greater dietary diversity. However, further study on habitat preference in all seasons is needed to further investigate this. In addition, the present study has also yielded new data supporting previously unrecorded Asian elephant preferences for *Thysanolaena maxima, Sterculia villosa, Equisetum debile, Semecarpus anacardium* and *Hypericum uralum.*

## Conclusion

Asian elephants have a diverse diet including monocot and dicot plants. Their diet in the dry season (February–April) contained a higher proportion of dicots compared to that of the wet season (June–September). There was a negative correlation between availability of plants and preference by elephants, suggesting food selection by elephants is not passively driven by relative availability, but related to specific preferences [44]. Further studies are needed to understand this feeding selectivity and its implications for the elephants. The current study provides baseline information about different types of natural food available in the Parsa and Chitwan regions of Nepal, and their relative importance in the diets of elephants in and around the PWR and CNP. This information is important for realising successful outcomes for the conservation of Asian elephants and improved seasonal management for the long-term protection of this endangered species and its shrinking habitat.

## Additional file

**Additional file 1: Appendix** Species, family, type of plant and plant parts consumed, and preference index for the majority of plants consumed by wild Asian elephants. A preference index score >1 indicates a food that was utilised proportionately more than its occurrence in the environment, and food with a preference index score <1 was utilised proportionately less than its occurrence in the environment.
