# Erratum to: Feeding preferences of the Asian elephant (*Elephas maximus*) in Nepal

**DOI:** 10.1186/s12898-016-0110-z

**Published:** 2016-12-07

**Authors:** Raj Kumar Koirala, David Raubenheimer, Achyut Aryal, Mitra Lal Pathak, Weihong Ji

**Affiliations:** 1 Human Wildlife Interaction Research Group, Institute of Natural and Mathematical Sciences, Massey University, Private Bag 102 904, Albany, Auckland, 0745 New Zealand; 2 Institute of Forestry, Tribhuvan University, Pokhara, Nepal; 3 The Charles Perkins Centre and School of Life and Environmental Sciences, University of Sydney, Sydney, NSW Australia; 4 Department of Forestry & Resource Management, Toi Ohomai Institute of Technology, Rotorua, 3046 New Zealand; 5 Waste Management NZ Ltd, Auckland, New Zealand; 6 Department of Plant Resources, National Herbarium and Plant Laboratories, Godawari, Nepal

## Erratum to: BMC Ecol (2016) 16:54 DOI 10.1186/s12898-016-0105-9

After publication of the original article [1], it was brought to our attention that the phrase ‘fifty-seven species of plants in 25 families were found to be eaten by Asian elephants, including 12 species of grasses, five shrubs, two climbers, one herb and 37 species of trees’ within the “Abstract” section on page 1 should read ‘fifty-seven species of plants in 28 families were found to be eaten by Asian elephants, including 13 species of grasses, five shrubs, two climbers, one herb and 36 species of trees’.

The phrase ‘in total, 57 species of plants (13 grass, five shrubs, two climbers, one herb and 36 tree species) belonging to 27 taxonomic families were eaten by Asian elephants’ on page 5 in the “Results” section should read ‘in total, 57 species of plants (13 grass, five shrubs, two climbers, one herb and 36 tree species) belonging to 28 taxonomic families were eaten by Asian elephants’.

In addition, the phrase ‘the present study recorded 57 plant species within 25 families that were foraged by Asian elephants in the PWR and CNP’ on page 6 in the “Discussion” section should read ‘the present study recorded 57 plant species within 28 families that were foraged by Asian elephants in the PWR and CNP’.

The phrases have been updated in the original article, and published in this erratum for quick reference. We apologise for any confusion this may have caused.
