# Supplementary material for: Feeding preferences of the Asian elephant (Elephas maximus) in Nepal
Source: BMC Ecol. 2016 Nov 17;16:54. doi: 10.1186/s12898-016-0105-9 (PMC5114758; doi:10.1186/s12898-016-0105-9)
Supplement: Supplementary file 1 — Additional file 1: Appendix Species, family, type of plant and plant parts consumed, and preference index for the majority of plants consumed by wild Asian elephants. A preference index score >1 indicates a food that was utilised proportionately more than its occurrence in the environment, and food with a preference index score <1 was utilised proportionately less than its occurrence in the environment. [file 12898_2016_105_MOESM1_ESM.docx]

**Appendix**

**Parsa Wet Season**

| **Plant Species** | **Type** | **Family** | **Parts Eaten** | **IVI** | **Utilisation%** | **Preference** |
| --- | --- | --- | --- | --- | --- | --- |
| *Acacia catechu* | Tree | Mimosaceae | Leaves, twigs and bark | 16.38 | 2.8 | 0.17 |
| *Asparagus racemosus* | Under Shrub | Asparagaceae | Leaves, twigs and stem | 9.04 | 0.2 | 0.02 |
| *Bauhinia purpurea L.* | Tree | Fabaceae | Leaves and twigs | 5.13 | 0.1 | 0.02 |
| *Bamboosa spp.* | Grass | Poaceae | Leaves and twigs | 1.33 | 3.5 | 2.63 |
| *Bauhinia vahlii* | Climber | Fabaceae | Leaves and twigs | 5.47 | 0.1 | 0.02 |
| *Bombax ceiba* | Tree | Malvaceae | Leaves and bark | 2.74 | 2.85 | 1.04 |
| *Bridelia retusa* | Tree | Euphorbiaceae | Leaves and twigs | 2.3 | 0.2 | 0.09 |
| *Careya arborea* | Tree | Lecythidaceae | Leaves and twigs | NA | NA | NA |
| *Casaris eliptica* | Tree | Salicaceae | Leaves and twigs | 7.08 | 0.05 | 0.01 |
| *Sida rhombifolia* | Shrub | Malvaceae |  | 3.05 | 2.24 | 0.73 |
| *Cymbopogan spp.* | Grass | Poaceae | Stem with leaves | 51.35 | 0.2 | 0.00 |
| *Dalbergia sissoo* | Tree | Fabaceae | Leaves, twigs and bark | 1.11 | 0.7 | 0.63 |
| *Digitaria ciliaris* | Grass | Poaceae | Stem with leaves | 20.41 | 0.91 | 0.04 |
| *Dillenia pentagyna* | Tree | Dilleniaceae | Leaves and twig | 48.61 | 11.32 | 0.23 |
| *Duabanga sonneratioides* | Tree | Lythraceae |  | 0.92 | 0.2 | 0.22 |
| *Equisetum debile* | Herb | Equisetaceae | Leaves and stem | 0.92 | 1.82 | 1.98 |
| *Ficus semicordata* | Tree | Moraceae | Leaves | 2.94 | 0.6 | 0.20 |
| *Garuga pinnata* | Tree | Burseraceae | Stem and bark | 11.59 | 17.59 | 1.52 |
| [*Hemarthria compressa*](https://www.google.co.nz/search?hl=en&authuser=0&biw=1366&bih=648&q=Hemarthria+compressa&spell=1&sa=X&ved=0ahUKEwjMtK2wk5DQAhUCG5QKHdgqBcoQvwUIFigA) | Grass | Poaceae | Stem with leaves | 38.87 | 0.1 | 0.00 |
| *Holarrhena pubescens* | Tree | Euphorbiaceae | Leaves and twig | 1.78 | 8.08 | 4.54 |
| *Hypericum uralum* | Shrub | Hypericaceae |  | 2.02 | 2.39 | 1.18 |
| *Hypoxis aurea* | Grass | Poaceae | Stem with leaves | 3.89 | 7.28 | 1.87 |
| *Lagerstroemia parviflora* | Tree | Lythraceae | Leaves and bark | 22.55 | 0.8 | 0.04 |
| *Leea robusta* | Large shrub/Tree | Leeaceae | Leaves | 0.85 | 1.51 | 1.78 |
| *Litsea monopetala* | Tree | Lauraceae | Leaves | 1.8 | 0.4 | 0.22 |
| *Mallotus philippensis* | Tree | Euphorbiaceae | Leaves | 10.36 | 1.61 | 0.16 |
| *Musa spp.* | Tree | Musaceae | Leaves and stem | 1.82 | 0.3 | 0.16 |
| *Osyris lanceolata/Wightiana* | Tree | Santalaceae |  | 1.34 | 0.05 | 0.04 |
| *Desmodium oojeinense* | Tree | Fabaceae | Bark | 0.87 | 0.4 | 0.46 |
| *Paspalum spp.* | Grass | Poaceae | Stem with leaves | 2.31 | 3 | 1.30 |
| *Phoenix humilis* | Shrub | Palmae | Leaves, fruit and root | 2.61 | 0.3 | 0.11 |
| *Phragmites karka* | Grass | Poaceae | Stem with leaves | 3.35 | 1.51 | 0.45 |
| *Saccharum bengalensis* | Grass | Poaceae | Stem with leaves | 2.34 | 3 | 1.28 |
| *Saccharum spontaneum* | Grass | Poaceae | Stem with leaves | 36.92 | 0.4 | 0.01 |
| *Shorea robusta* | Tree | Dipterocarpaceae | Bark | 32.98 | 0.4 | 0.01 |
| *Spatholobus parviflorus* | Climber | Fabaceae | Leaves, bark and stem | 4.76 | 17.79 | 3.74 |
| *Sterculia villosa* | Tree | Malvaceae | Leaves and Bark | 6.11 | 4.5 | 0.74 |
| *Terminalia chebula* | Tree | Combretaceae | Bark | 1.14 | 0.1 | 0.09 |
| *Thysanolaena maxima* | Grass | Poaceae | Leaves and twigs | 1.32 | 7.28 | 5.52 |
| *Ziziphus mauritiana* | Tree | Rhamnaceae | Leaves and twigs | 2.73 | 0.11 | 0.04 |

**Chitwan Wet season**

| **Plant species** | **Type** | **Family** | **Parts Eaten** | **IVI** | **Utilisation %** | **Preference** |
| --- | --- | --- | --- | --- | --- | --- |
| *Artocarpus heterophyllus* | Tree | Moraceae | Leaves, fruits | NA | NA | NA |
| *Bauhinia purpurea L.* | Tree | Fabaceae | Leaves and twigs | NA | NA | NA |
| *Bamboosa spp.* | Grass | Poaceae | Stem, leaves and twigs | 6.46 | 5.95 | 0.92 |
| *Bridelia retusa* | Tree | Euphorbiaceae | Leaves and twigs | 1.03 | 1.48 | 1.44 |
| *Careya arborea* | Tree | Lecythidaceae | Leaves ,twigs and bark | NA | NA | NA |
| *Sida rhombifolia* | Shrub | Malvaceae |  | 1.22 | 0.12 | 0.10 |
| *Cleistocalyx operculata* | Tree | Myrtaceae | Leaves and twigs | 25.46 | 3.97 | 0.16 |
| *Cymbopogan spp.* | Grass | Poaceae | Stem with leaves | 1.14 | NA | NA |
| *Desmodium oojeinense* | tree | Fabaceae |  | 2.23 | 0.49 | 0.22 |
| *Desmostachya bipinnata* | Grass | Poaceae | Stem with leaves | 3.56 | 3.56 | 1.00 |
| *Dillenia pentagyna* | Tree | Dilleniaceae | Leaves and twigs | 15.85 | 1.98 | 0.12 |
| *Duabanga sonneratioides* | Tree | Lythraceae |  | 2.19 | 0.24 | 0.11 |
| *Ficus hispida* | Tree | Moraceae | Leaves and twigs | NA | NA | NA |
| *Ficus religiosa* | Tree | Moraceae | Leaves and twigs | NA | 4.76 |  |
| *Hemarthria compressa* | Grass | Poaceae | Stem with leaves | 9.87 | 0.74 | 0.07 |
| *Holarrhena pubescens* | Tree | Euphorbiaceae | Leaves and twig | 2.04 | 0.49 | 0.24 |
| *Imperata cylindrica* | Grass | Poaceae | Stem with leaves | 52.18 | 1.31 | 0.03 |
| *Leea macrophylla* | Shrub/Tree | Leeaceae |  | 3.04 | 0.37 | 0.12 |
| *Litsea monopetala* | Tree | Lauraceae | Leaves | 17.17 | 12.4 | 0.72 |
| *Mallotus philippensis* | Tree | Euphorbiaceae | Leaves | 3.99 | 9.9 | 2.48 |
| *Murrya coenigii* | Tree | Rutaceae | Leaves and twigs | 4.92 | 0.49 | 0.10 |
| *Myrsine semiserrata* | Tree | Myrsinaceae | Bark | NA | NA |  |
| *Phragmites karka* | Grass | Poaceae | Stem with leaves | 2.62 | 6.62 | 2.53 |
| *Premna integrifolia L.* | Tree | Verbenaceae |  | 1.03 | 0.19 | 0.18 |
| *Saccharum bengalensis* | Grass | Poaceae | Stem with leaves | 3.86 | 0.37 | 0.10 |
| *Saccharum spontaneum* | Grass | Poaceae | Stem with leaves | 12.29 | 6.75 | 0.55 |
| *Shorea robusta* | Tree | Dipterocarpaceae | Bark | 131.73 | 23.44 | 0.18 |
| *Spatholobus parviflorus* | Climber | Fabaceae | Leaves, bark and stem | 4.08 | 37.22 | 9.12 |
| *Syzygium cumini* | Tree | Myrtaceae | Leaves, twigs and bark | 1.5 | 0.25 | 0.17 |

**Parsa Dry season**

| **Plant species** | **Type** | **Family** | **Parts Eaten** | **IVI** | **Utilisation %** | **Preference** |
| --- | --- | --- | --- | --- | --- | --- |
| *Acacia catechu* | Tree | Mimosaceae | Bark | 37.04 | 7.92 | 0.21 |
| *Cymbopogan spp* | Grass | Poaceae | Stem with leaves | 6.38 | 14.85 | 2.33 |
| *Duabanga sonneratioides* | Tree | Lythraceae |  | 13.67 | 1.98 | 0.14 |
| *Lagerstroemia parviflora* | Tree | Lythraceae | Leaves and bark | 2.11 | 5.94 | 2.82 |
| *Litsea monopetala* | Tree | Lauraceae | Leaves | 1.80 | 1.98 | 1.10 |
| *Mallotus philippensis* | Tree | Euphorbiaceae | Leaves, bark | 20.37 | 11.88 | 0.58 |
| *Phoenix humilis* | Shrub | Palmae | Leaves, fruits and root | 3.40 | 9.90 | 2.91 |
| *Saccharum spontaneum* | Grass | Poaceae | Stem with leaves | 2.60 | 9.90 | 3.81 |
| *Shorea robusta* | Tree | Dipterocarpaceae | Bark | 40.00 | 1.98 | 0.05 |
| *Spatholobus parviflorus* | Climber | Fabaceae | Leaves, bark, and stem | 1.70 | 15.84 | 9.32 |

**Chitwan Dry Season**

| **Plant species** | **Type** | **Family** | **Parts Eaten** | **IVI** | **Utilisation %** | **Preference** |
| --- | --- | --- | --- | --- | --- | --- |
| *Caesalpinia spp.* | Tree | Fabaceae | Leaves and flower | 3.36 | NA | NA |
| *Ficus hirta* | Tree | Moraceae | Leaves and twigs | 1.25 | NA | NA |
| *Ficus racemosa* | Tree | Moraceae | Bark | 1.26 | 11.63 | 9.23 |
| *Ficus semicordata* | Tree | Moraceae | Leaves and Bark | 7.9 | 7.75 | 0.98 |
| *Mallotus philippensis* | Tree | Euphorbiaceae | Bark | 1.26 | 9.30 | 7.38 |
| *Miliusa velutina* | Tree | Annonaceae | Leaves, bark | 4.22 | 6.20 | 1.47 |
| *Narenga porphyrocoma* | Grass | Poaceae | Stem with leaves | 5.8 | 1.55 | 0.27 |
| *Phoenix humilis* | Shrub | Palmae | Leaves, fruits and root | 1.25 | 1.55 | 1.24 |
| *Phragmites karka* | Grass | Poaceae | Stem with leaves | 3.09 | 3.10 | 1.00 |
| *Saccharum bengalensis* | Grass | Poaceae | Stem with leaves | 7.82 | 13.95 | 1.78 |
| *Saccharum spontaneum* | Grass | Poaceae | Stem with leaves | 37.3 | 12.40 | 0.33 |
| *Semecarpus anacardium* | Tree | Anacardiaceae | Leaves and bark | 1.39 | 9.30 | 6.69 |
| *Spatholobus parviflorus* | Climber | Fabaceae | Leaves, bark and stem | 6.96 | 10.85 | 1.56 |
| *Cleistocalyx operculata* | Tree | Myrtaceae | Leaves and twigs | 1.3 | 4.65 | 3.58 |
